# No male mate choice for female boldness in a bi-parental West African cichlid, the rainbow krib (*Pelvicachromis pulcher*)

**DOI:** 10.7717/peerj.5373

**Published:** 2018-08-09

**Authors:** Ulrike Scherer, Wiebke Schuett

**Affiliations:** 1 Institute of Zoology, Universität Hamburg, Hamburg, Germany; 2 School of Life Sciences, University of Sussex, Brighton, United Kingdom

**Keywords:** Activity, Assortment, Behavioural consistency, Behavioural level, Behavioural stability, Mating preference, Personality, Predation risk, Sexual selection, Similarity

## Abstract

**Background:**

In many species, males have a lower reproductive investment than females and are therefore assumed to increase their fitness with a high number of matings rather than by being choosy. However, in bi-parental species, also males heavily invest into reproduction. Here, reproductive success largely depends on costly parental care; with style and amount of parental effort in several cases being associated with personality differences (i.e., consistent between-individual differences in behaviour). Nonetheless, very little is known about the effect of personality differences on (male) mate choice in bi-parental species.

**Methods:**

In the present study, we tested male mate choice for the level and consistency of female boldness in the rainbow krib, *Pelviachromis pulcher*, a bi-parental and territorial West African cichlid. Individual boldness was assumed to indicate parental quality because it affects parental defence behaviour. For all males and females, boldness was assessed twice as the activity under simulated predation risk. Mate choice trials were conducted in two steps. First, we let a male observe two females expressing their boldness. Then, the male could choose between these two females in a standard mate choice test.

**Results:**

We tested for a male preference for behavioural (dis-)similarity vs. a directional preference for boldness but our data support the absence of effects of male and/or female boldness (level and consistency) on male mating preference.

**Discussion:**

Our results suggest female personality differences in boldness may not be selected for via male mate choice.

## Introduction

Ever since Darwin, female mate choice has received extensive attention in sexual selection studies though male mate choice has long been overlooked (Arnaud & Haubruge, 1998; Herdman, Kelly & Godin, 2004). Males were assumed not to be choosy because of their low reproductive investment: the production of tiny sperm is less costly than the production of large oocytes allowing males to increase their fitness through a high number of matings rather than through choosiness (Bateman, 1948; Trivers, 1972; Kokko & Jennions, 2003). However, male investment into reproduction is not as low as previously presumed; the production of sperm, especially when produced in a large amount, can actually be quite costly (Olsson, Madsen & Shine, 1997; Wedell, Gage & Parker, 2002; Caballero-Mendieta & Cordero, 2013). Furthermore, characteristics of the mating system can lead to an equal or even heavily male-biased reproductive investment, such as in bi-parental and sex-role reversed species (Gross & Sargent, 1985; Svensson, 1988; Cantoni & Brown, 1997). In bi-parental species, both the male and the female parent provide intensive offspring care, which can be extremely costly (Marconato, Bisazza & Fabris, 1993; Steinhart et al., 2004; Royle, Smiseth & Kölliker, 2012). Under such increased costs of reproduction (e.g., time, energy and resources) not only females but also males are expected to be choosy (Bonduriansky, 2001; Wong & Jennions, 2003).

Male mating preferences have largely been tested for female traits that indicate fecundity (Bonduriansky, 2001; Edward & Chapman, 2011; Wang et al., 2017); for instance body size (Olsson, 1993), weight (Welke, Zimmer & Schneider, 2012), fatness (Bonduriansky & Brooks, 1998) or colouration (Amundsen & Forsgren, 2001). Little is known about male mating preference for consistent differences in behavioural traits (e.g., aggression, boldness and explorative tendency), also referred to as personality differences, coping styles or temperaments (Schuett, Tregenza & Dall, 2010). To the best of our knowledge, the relatively few studies examining mate choice for personalities mainly consider female but not male mate choice (Schuett, Godin & Dall, 2011a; Kralj-Fišer et al., 2013; Teyssier et al., 2014; Montiglio et al., 2016; Scherer, Kuhnhardt & Schuett, 2017a; but see Laubu et al., 2017). Male mate choice for personality traits is especially interesting in bi-parental species because (I) female behaviour can directly affect reproductive success through amount and style of parental care (reviewed in Chira, 2014). For example, female exploratory behaviour increased the number of fledglings in blue tits, *Cyanistes caeruleus* (Mutzel et al., 2013) and aggressive Ural owl females, *Strix uralensis*, raised more offspring (Kontiainen et al., 2009). Further, (II) due to the mutual provision of care also the interplay between male and female personality has the potential to affect reproductive success (Schuett, Dall & Royle, 2011b; David et al., 2015; Laubu et al., 2016). Behavioural similarity in the level and consistency of exploratory behaviour positively affected fledgling condition of breeding pairs in the zebra finch, *Taeniopygia guttata* (Schuett, Dall & Royle, 2011b). In the convict cichlid, *Amatitlania siquia*, pairs that achieved post-pairing similarity on the proactive-reactive continuum could increase the number of their offspring (Laubu et al., 2016).

In the present study, we tested male mating preference for female boldness (probability to engage into risky behaviour; Wilson et al., 1994) in a territorial and bi-parental West African cichlid species, the rainbow krib, *Pelvicachromis pulcher*. Bi-parental cichlids commonly show a division of labour with specific sex roles during offspring care (e.g., Itzkowitz, 1984; Lavery & Reebs, 1994; McKaye & Murry, 2008): typically, males do a greater proportion of the territory defence (vigilance behaviours, attacking intruders) females provide more direct offspring care (egg oxygenation, keeping the brood together, guidance to feeding grounds). Accordingly, one could hypothesize females to show a directional preference for male boldness (indicating high parental quality). In contrast, males could be expected to show no preference for female boldness because the benefit of a high behavioural level in female boldness during direct offspring care might be rather low. However, we previously tested female preference for male boldness in this species (Scherer, Kuhnhardt & Schuett, 2017a) and found a dis-assortative preference for the behavioural level and an assortative preference for the consistency of male boldness. Most importantly, (dis-)assortment indicates mutual mate choice because it results from a joint assessment process (Johnstone, 1997). Thus, not only females but also males might choose their mate on the basis of its boldness in the rainbow krib. Such a preference pattern may ease parental care coordination through a facilitation of labour division with the bold parent performing territory defence and the shy parent providing direct offspring care. That is, roles might be based on individual behavioural predisposition rather than on the sex (Scherer, Kuhnhardt & Schuett, 2017a). Here, we used an experimental design similar to our female choice study testing for the male perspective: males were allowed to choose between two females that differed in their level and consistency of boldness (activity under simulated predation risk). Prior to mate choice, males were allowed to eavesdrop on female boldness. We measured individual boldness twice to determine behavioural consistency at the individual and population level. We hypothesized to find the same pattern as in our female choice study (Scherer, Kuhnhardt & Schuett, 2017a): consistent personality differences in both sexes and a mating preference for a dissimilar level and similar consistency of boldness (II). Alternatively, we considered female behaviour itself to be important (I): we tested for a general male preference for a high level and high consistency of female boldness. A high level of boldness could indicate high parental effort, while behavioural consistency could indicate the reliability of the trait and, therefore, the quality of the signal (Royle, Schuett & Dall, 2010).

## Materials & Methods

### Study animals and holding conditions

All fish were kept at the Universität Hamburg (100 × 50 × 25 cm tanks, 26 ± 1 °C water temperature, aerated and filtered water, weekly water changes, 12:12 h light:dark). Male *P. pulcher* originated from the university breeding stock but due to a heavily skewed sex ratio females were largely bought as juveniles from external suppliers. Fish were held in shoals of approx. Forty individuals matched for sex and origin (university stock: matched for family; external suppliers: matched for supplier and batch). Fish were fed 5 days a week with live *Artemia spp*.

For the duration of experimental trials fish were transferred to individual housing tanks (25 × 50 × 25 cm; same holding conditions as above) and were fed 7 days a week ensuring equal conditions between successive trials. On experimentation days, fish were fed after the observations. All fish were measured for their standard length (males: mean ± SE = 5.03 ± 0.08 cm; females: mean ± SE = 3.97 ± 0.04 cm) using ImageJ (Schneider, Rasband & Eliceiri, 2012) 5 days before experimental trials and were marked for individual identification using VIE tags (visible implant elastomers; VIE-Northwest Marine Technology, Shaw Island, WA, USA) four days before experimental trials. Such VIEs do not affect mate choice in *P. pulcher* (Schuett et al., 2017). After VIE tagging, all individuals resumed to normal behaviour without any signs of distress within less than 24 h.

### General outline

Experimental trials were conducted during July and August 2017. Our work was approved by the German “Behörde für Gesundheit und Verbraucherschutz Hamburg” (permission number 52/16). We used a similar experimental set up and procedure as described in Scherer, Kuhnhardt & Schuett (2017a). In order to assess the level and consistency of boldness, all males (*N* = 44) and females (*N* = 44) were tested for their boldness twice (please see *‘Boldness test’*) with 3 days in between; successive trials were performed on the same time of day (±15 min). We always boldness typed two same-sex individuals simultaneously (with no visual contact between test fish). During female boldness tests, males were allowed to observe female behaviour. Male mating preference for the two females was tested directly after the female boldness test in a standard binary choice test (please see *‘Mate choice trials’*). Such binary choice tests are a standard procedure being appropriate to predict mating preferences in cichlid fishes from the time spent near potential mates (Thünken et al., 2007; Dechaume-Moncharmont et al., 2011; Scherer, Kuhnhardt & Schuett, 2017a). Importantly, male choice was assessed after and not during predator exposure reducing potential effects of male anti-predator behaviour on male mate choice. Empirical studies have shown that fish observe (and remember) conspecific behaviour, and that they later use such information during their own social interactions with the previously observed individual (Schlupp, Marler & Ryan, 1994; Doutrelant & McGregor, 2000; Witte & Godin, 2010; Bierbach et al., 2013; Scherer, Kuhnhardt & Schuett, 2017a). Male preference was assessed for each male once (*N* = 44). Each female dyad (*N* = 22) was used for two mate choice trials, once after each boldness test. We performed a complete water change in all experimental tanks before each boldness test/mate choice trial.

### Boldness test

Boldness was measured as the individual activity under simulated predation risk (hereafter APR; Scherer, Kuhnhardt & Schuett, 2017a; Scherer, Godin & Schuett, 2017b) via exposing individuals to a video animated photograph of a naturally occurring predator, the African obscure snakehead, *Parachanna obscura* (*N* = 4, mean ± SE standard length = 16.11 ± 0.38 cm). Predator specimen were animated to swim back and forth in front of a white background using PowerPoint (1 cm/sec) (Scherer, Kuhnhardt & Schuett, 2017a; Scherer, Godin & Schuett, 2017b). Rainbow kribs decrease their activity in the presence of such animated predators compared to predator free control trials (Scherer, Godin & Schuett, 2017b). Further, this response is comparable to the individual response towards a live *P. obscura* specimen (Scherer, Godin & Schuett, 2017b).

**Figure 1:**
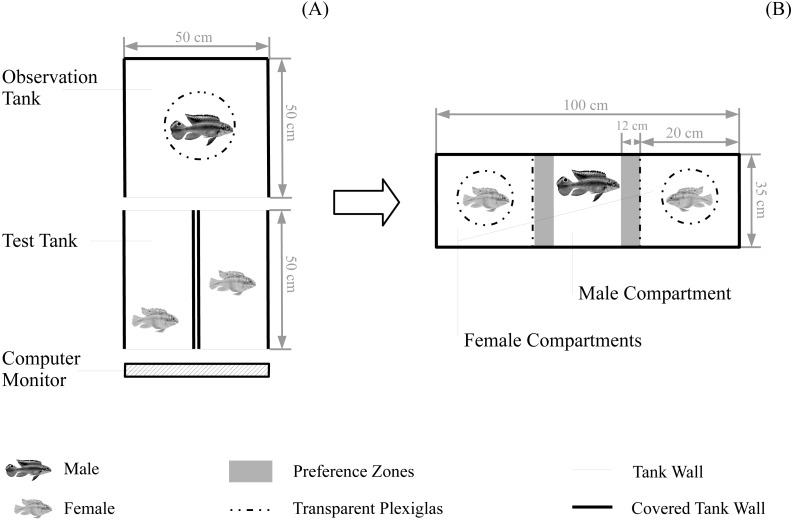
Experimental set-up for behavioural tests. Set-up for (A) the boldness test and for female boldness tests (B) the subsequent mate choice test. Water level for all tanks was 10 cm. Fish are not to scale.

To begin a boldness test, we introduced two same sex individuals into two neighbouring test tanks without visual contact (Fig. 1A). For boldness tests of males, simultaneously tested males were randomly chosen but for boldness tests of females, simultaneously tested females were matched for origin and standard length (size difference <5%; mean ± SE = 0.03 ± 0.01 cm). After an acclimation of 10 min, both test fish were allowed visual access to a computer monitor (UltraSharp U2412M 61 cm (24″); Dell, Round Rock, TX, USA) on one end of the two tanks through removal of a white separator. During this test period (duration = 11 min), we presented a randomly chosen animation of an unfamiliar predator specimen to both test fish. Also, we removed another white separator at the back of the two tanks for the duration of the test period allowing an observer fish (acclimated for 10 min) full view to both test fish and the predator animation (Fig. 1A). For female boldness tests, we randomly chose a male observer not being related (non-sibling and non-familiar) to the females for further assessment of male mating preference (please see ‘Mate choice trials’). For male boldness tests, we introduced a randomly chosen dummy female that was not part of this study. The observer fish was hidden in a cylinder (diameter = 20 cm), which was coated with one-way mirror foil. The usage of the cylinder ensured that both test fish were visible to the observer during the test period while the one-way foil reduced visibility of the observer to test fish (avoiding an impact of the observer on test fish behaviour). Observers did not show signs of distress when being kept in the cylinder. The observer tank was covered with black plastic plates, including a black plate covering the top to further decrease visibility of the observer to test fish. The sides of boldness test tanks were covered with white plastic plates to avoid disturbances and visual contact between test fish. Test periods were video-recorded from an above camera. After male boldness tests, test fish were returned to their individual housing tank. After female boldness tests, female test fish and the male observer were directly transferred to a mate choice chamber for assessing male mating preference (please see ‘Mate choice trials’).

Individual APR was assessed from the videos for all males and females as the total distance moved (cm) during 10 min (starting 1 min after the video start) using the animal tracking software Ethovision XT 11 (Noldus, Wageningen, The Netherlands). For all preference analyses, we used female APR of the boldness test that was observed by the respective observing male. For males, the individual behavioural level was assessed as the average APR of both boldness tests. Behavioural consistency was measured as inconsistency: the absolute value of the difference in the APR between the two boldness tests (Scherer, Kuhnhardt & Schuett, 2018). Due to an error in three male boldness tests (each trial including two simultaneously tested males) we had to remove six males from the data set. The two females of each boldness test were classified into bold (mean ± SE APR = 1,037.27 ± 113.24 cm moved) and shy (mean ± SE APR = 577.18 ± 79.26 cm moved), depending on their level of boldness relative to each other; and into consistent (mean ± SE inconsistency = 268.5 ± 40.5 cm) and inconsistent (mean ± SE inconsistency = 565.0 ± 60.2 cm), depending on their inconsistency relative to each other. Bold and shy females significantly differed in their level of behaviour (mean ± SE within-dyad difference in APR = 196.2 ± 34.0 cm moved; average over both female boldness tests used) (linear mixed-effect model with female behavioural level (APR in cm) as dependent variable, female level classification as fixed effect, and female ID as well as female dyad ID as random effects; ${\chi }_{1}^{2}=20.670$, *P* < 0.0001, coefficient ± SE = 450.6 ± 85.9 cm moved; *N* = 88 measures of 44 females in 22 dyads, each female tested twice). Likewise, consistent and inconsistent females significantly differed in their behavioural consistency (mean ± SE within-dyad difference in inconsistency = 296.6 ± 42.9 cm) (linear mixed-effect model with female inconsistency as dependent variable, female consistency classification as predictor variable, and female dyad ID as random effect; ${\chi }_{1}^{2}=16.434$, *P* < 0.0001, coefficient ± SE = 296.6 ± 60.0 cm; *N* = 22 female dyads). Importantly, the behavioural classification into bold and shy (or consistent and inconsistent) was based on the behavioural contrast between the two females of a dyad and does not represent a global classification.

### Mate choice trials

To begin a choice test, we transferred the two females and the observer male from the female boldness test tanks to the mate choice chamber (Fig. 1B): the male was transferred to the male compartment in the middle and the two females were randomly assigned to the two female compartments of the choice chamber. All fish were allowed to acclimate for 10 min without visual contact (removable white separators) followed by a 12 min test period with full visual contact between the three compartments (separators removed). Thereafter, we repeated this test period with the females being switched between the two female compartments controlling for a potential male side bias. All fish were allowed to acclimate without visual contact for 5 min before starting the second test period (duration = 12 min) with full vision. During the whole duration of mate choice trials, females were kept in Plexiglas cylinders (inner diameter = 7.4 cm) to control for general female locomotor activity. Prior to mate choice trials, we habituated females to the cylinders: we kept them in the cylinder for 45 min per day, on three consecutive days (starting 5 days before experimental trials, no cylinder training during the two days before the start of experimental trials). The mate choice chamber was surrounded with white plastic plates. Both test periods were video-recorded from above.

Male preference was assessed from the videos using Ethovision XT 11. We tracked the association time (sec); i.e., the amount of time spent near the two female compartments (within a zone-width of 10 cm, hereafter preference zone; Fig. 1B) during both test periods. Male preference for each female was then calculated over both test periods setting the total association time for one female into relation to the total association time for both females. This results into a preference score ranging from 0 (no time spent with a female) to 1 (100% of the total time spent with a female). Further, we calculated male side bias over the two test periods as the total amount of time spent in the left preference zone set into relation to the total amount of time spent in both preference zones (Scherer, Kuhnhardt & Schuett, 2017a). We *a priori* decided a male to be side-biased, when it spent more than 80% of the total association time in just one preference zone, regardless which female was there. Side-biased preference data were excluded from the analyses (e.g., Schlupp, Waschulewski & Ryan, 1999; Scherer, Kuhnhardt & Schuett, 2017a), (*N* = 3 excluded mate choice trials).

### Data analyses

Data were analysed in R version 3.4.0 (R Core Team, 2017). All data used for analyses are provided as supplemental information (Data S1 and S2). To assess behavioural consistency on population level, we calculated normal and adjusted (corrected for trial number) repeatabilities for male (*N* = 76 trials of 38 males) and female (*N* = 88 trials of 44 females) APR with 1,000 bootstrapping runs and 1,000 permutations using the *rptR*-package (Stoffel, Nakagawa & Schielzeth, 2017). Adjusted repeatabilities were calculated taking account for potential effects of habituation to the stimulus by adding the test trial number as fixed term (Bell, Hankison & Laskowski, 2009; Nakagawa & Schielzeth, 2010). Also, we tested for an effect of the boldness test trial number on APR in both sexes using paired *t*-tests.

In the present study, we tested for a linear function describing the relationship between male preference and female quality. Visual data inspection did not suggest a non-linear relationship. However, preference functions can also be shaped non-linearly (Wiegmann et al., 2013; Reinhold & Schielzeth, 2015). We tested for a directional male preference for a high level or high consistency of female boldness by running two linear mixed-effects models (LMMs) on male mating preference. As response variable, we used either male preference for bold females (*N* = 35) or for consistent females (*N* = 35), respectively. Female ID and female dyad ID were included as random effects but no fixed effects were included (aka null model). Deviation from random choice would be revealed when the 95% confidence interval (CI) of the intercept does not include 50% (Scherer, Kuhnhardt & Schuett, 2017a). In a different mate choice study, we found female rainbow kribs to prefer males that show a combination of high behavioural consistency and high level of aggression (Scherer, Kuhnhardt & Schuett, 2018). Therefore, we also tested males for a mating preference for females showing both high level and high consistency of boldness (*N* = 18) through running a third null model, again, only including female ID and female dyad ID as random effects.

We tested for a male preference for behavioural (dis-)similarity by fitting an LMM on male preference for bold females (*N* = 35). We included relative similarity in the behavioural level and relative similarity in the behavioural consistency as fixed effects and female ID as well as female dyad ID as random effects. Following Scherer, Kuhnhardt & Schuett (2017a), we calculated relative similarity as the male’s similarity with the shy female minus the male’s similarity with the bold female (for the level and consistency of behaviour, respectively). Similarity in the level and consistency of APR was calculated as the absolute value of the difference between the male and each of the two females, respectively. Relative similarity for the behavioural level was assessed using female behaviour shown during the respective male observation and average male behaviour shown over both boldness tests. Positive values of relative similarity indicate the male’s similarity with the bold female is higher than its similarity with the shy female, vice versa, negative values show the male’s similarity with the shy female is higher. Because male APR was strongly affected by the boldness test trial number (please see ‘Results’) we calculated two additional versions of relative similarity for the behavioural level; one version using male APR measured during the first boldness test, and another version using male APR measured during the second boldness test (again, we used female APR that was observed by the respective male, not the average female APR). We performed the above described model three times; all models were identical but contained different versions of relative similarity for the behavioural level (calculated using male APR assessed either during the first-, the second- or both boldness tests). Prior to analyses, male preference score was arcsine-square root-transformed for normality of residuals and predictor variables (relative similarity in the behavioural level and in behavioural consistency) were z-transformed for standardisation. We report partial R^2^ with 95% confidence levels (CL), calculated using the *r2glmm*-package (Jaeger, 2016), and estimates for all predictor variables. For insignificant predictors we report test statistics derived from the latest model incorporating the term (backward model selection). Model assumptions were visually checked. For an example code of our preference analyses please see Scherer, Kuhnhardt & Schuett (2018).

Differences in the behavioural contrast between the two females of a dyad (that is how much the females differed in their level and consistency of behaviour, respectively) are inherent in our experimental design because female dyads were only matched for size but formed randomly in regard to their behaviour. We tested for an effect of female behavioural contrast on male mate choice by fitting an LMM on male choosiness (absolute value of the difference in male strength of preference for the two females of a dyad) (*N* = 35). We included female within-dyad contrast in the behavioural level as well as female within-dyad contrast in behavioural consistency as fixed effects and female dyad ID as random effect. Female within-dyad contrast in the behavioural level did not affect male choosiness (LMM: ${\chi }_{1}^{2}=1.059$, *P* = 0.303, coefficient ± SE (standardised) = − 0.051 ± 0.048; *R*^2^ = 0.032, 95% CL [0.000–0.229]; *N* = 35). However, male choosiness increased with increasing female within-dyad contrast in behavioural consistency (LMM: ${\chi }_{1}^{2}=5.703$, *P* = 0.017, coefficient ±SE (standardised) = 0.137 ± 0.054; *R*^2^ = 0.202, 95% CL [0.027–0.451]; *N* = 35). Also, we tested whether male choosiness (*N* = 35) was affected by the relative similarity in the level (male average APR used for calculation) and consistency of boldness by fitting another LMM on male choosiness, including relative similarity in the behavioural level (absolute value) as well as relative similarity in the behavioural consistency (absolute value) as fixed effects and female dyad ID as random effect. We did not detect any effects of relative similarity in the level (LMM: ${\chi }_{1}^{2}=1.441$, *P* = 0.230, coefficient ± SE (standardised) = − 0.063 ± 0.047; *R*^2^ = 0.042, 95% CL [0.000–0.250]; *N* = 35) or consistency (LMM: ${\chi }_{1}^{2}=2.114$, *P* = 0.146, coefficient ± SE (standardised) = 0.078 ± 0.051; *R*^2^ = 0.067, 95% CL [0.000–0.291]; *N* = 35) of boldness on male choosiness.

Even though there was not much suggestive evidence for the behavioural contrast within dyads affecting male choosiness, we performed all preference analyses (testing for a directional preference and testing for male choice based on (dis-)similarity) with the full data set and with a smaller data set where the trials with low behavioural contrast were removed. For the directional preference analyses, we removed all preference data derived from mate choice trials where female within-dyad behavioural contrast in the level (*N* = 15 trials removed) or consistency (*N* = 17 trials removed) was less than 200 cm moved. When testing for male preference for high level and high consistency females we used the sum of the behavioural contrast in level and consistency as threshold (again 200 cm moved; *N* = 24 trials removed). Similarly, for our preference analysis regarding mate choice for (dis-) similarity, we removed all mate choice trials with relative similarity in level and consistency (absolute values added up; *N* = 11 trials removed) being less than 200 cm moved. The threshold of 200 cm was chosen to ensure a minimum behavioural contrast without decreasing *N* (and the statistical power) too much (please note, we obtained qualitatively the same results when other thresholds were chosen).

## Results

**Figure 2:**
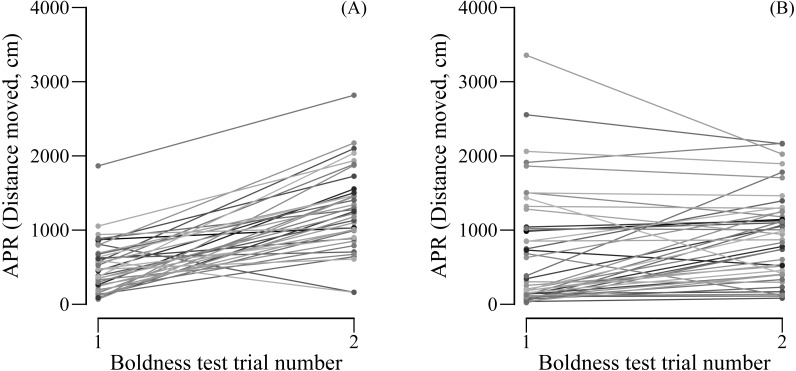
(A) Male and (B) female APR (activity under simulated predation risk) over two boldness tests.

We found female (LMM: *R* = 0.673, SE = 0.090, 95% CI [0.448–0.808], *N* = 44) but not male APR (LMM: *R* = 0.000, SE = 0.088, 95% CI [0.000–0.273], *N* = 38) to be repeatable over the two boldness tests. However, when controlling for the trial number, both females (LMM: *R* = 0.707, SE = 0.082, 95% CI [0.515–0.837], *N* = 44) and males (LMM: *R* = 0.338, SE = 0.137, CI = [0.086–0.590], *N* = 38) were significantly repeatable in their boldness. Male boldness significantly increased from the first (mean ± SE APR = 498.2 ± 57.8 cm moved) to the second (mean ± SE APR = 1265.8 ± 89.4 cm moved) boldness test (paired *t*-test: *t*_37_ = −8.861, *P* < 0.0001, *N* = 38; Fig. 2A). Although less pronounced, also female boldness increased from the first (mean ± SE APR = 703 ± 86.8 cm moved) to the second (mean ± SE APR = 911.4 ± 116.3 cm) boldness test (paired *t*-test: *t*_43_ = −2.650, *P*= 0.011, *N* = 44; Fig. 2B).

**Figure 3:**
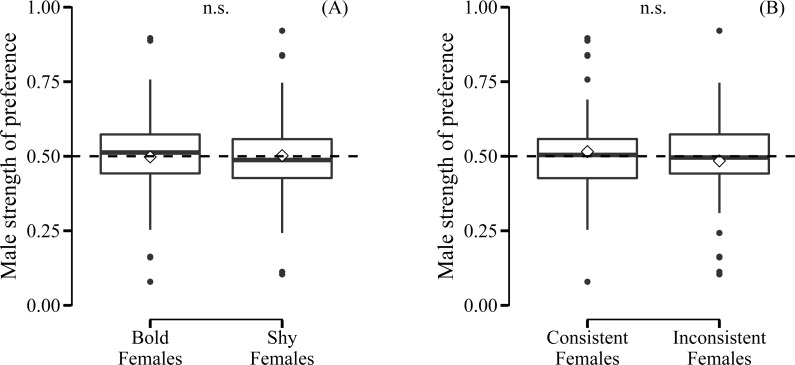
Male preference for the (A) level and (B) consistency of female boldness. Boldness was measured as APR (activity under simulated predation risk; in cm). Boxplots with 1.5 interquartile ranges, mean (−) and medians (◊); n.s. = non-significant. No deviation from random choice (male strength of preference = 0.50, dashed line) detected.

**Figure 4:**
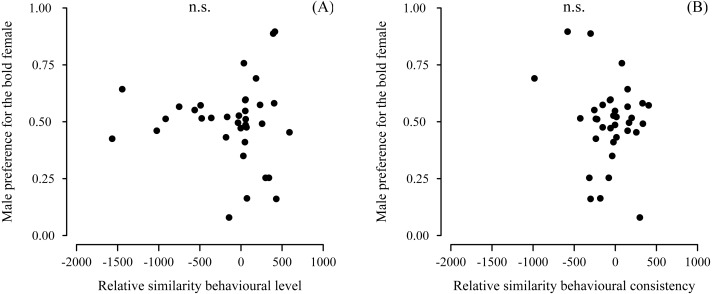
Male preference for the relative similarity in the (A) level and (B) consistency of female boldness. Boldness was measured as APR (activity under simulated predation risk; in cm). Relative similarity in average male APR and female APR observed directly before mate choice. Positive values of relative similarity indicate the male’s similarity with the respective bold female of a female dyad was higher than its similarity with the respective shy female. Vice versa: negative values indicate the male was more similar to the shy female than to the bold female. No significant effects detected (n.s., non-significant).

Male preference for bold females did not show a deviation from random choice (mean preference: 0.497; 95% CI [0.432–0.562], *N* = 35) (Fig. 3A). Although male choosiness increased with increasing behavioural contrast in female consistency (please see ‘Data analyses’), male preference for consistent females did not deviate from random choice (mean preference: 0.519; 95% CI [0.446–0.593], *N* = 35) (Fig. 3B). Likewise, male preference for females that were both bold and consistent did not deviate from random choice (mean preference: 0.478; 95% CI [0.409–0.548], *N* = 35). Furthermore, we did not detect any effects of relative similarity in the level or consistency of APR on male mating preference for bold females (Table 1, Fig. 4). Also, when performing our preference analyses considering the effect of the boldness test trial number on male APR, and using a smaller data set where mate choice trials with a low behavioural contrast in absolute or relative female behaviour were removed, we did not detect significant effects of female boldness on male mate choice (Table 1).

**Table 1:** Summary of LMM analyses of male choice for (dis-)similarity in boldness. Boldness was measured as APR (activity under simulated predation risk; in cm). All LMMs had female ID and female dyad ID as random effects. Models were based either on the full data set or a reduced data set where all mate choice trials with relative similarity in the level and consistency of behaviour smaller than 200 cm moved (absolute value of the sum) were removed.

Data set	Male behavioural level	Dependent variable	Fixed effects	Estimate ± SE	*χ*^2^	*P*	R ^2^ [CL]	N
Full data set	First boldness test	Male preference	Relative similarity level	−0.037 ± 0.032	1.311	0.252	0.038 [0.000, 0.241]	35
			Relative similarity consistency	−0.041 ± 0.032	1.618	0.203	0.046 [0.000, 0.257]	
	Second boldness test	Male preference	Relative similarity level	0.012 ± 0.032	0.139	0.709	0.004 [0.000, 0.155]	35
			Relative similarity consistency	−0.041 ± 0.032	1.618	0.203	0.046 [0.000, 0.257]	
	Mean	Male preference	Relative similarity level	−0.007 ± 0.032	0.059	0.808	0.002 [0.000, 0.146]	35
			Relative similarity consistency	−0.041 ± 0.032	1.618	0.203	0.046 [0.000, 0.257]	
Low behavioural contrast removed	First boldness test	Male preference	Relative similarity level	0.010 ± 0.033	0.095	0.757	0.003 [0.000, 0.320]	15
			Relative similarity consistency	−0.078 ± 0.041	3.247	0.072	0.163 [0.001, 0.556]	
	Second boldness test	Male preference	Relative similarity level	−0.004 ± 0.032	0.016	0.901	0.001 [0.000, 0.314]	15
			Relative similarity consistency	−0.015 ± 0.032	0.235	0.628	0.017 [0.000, 0.355]	
	Mean	Male preference	Relative similarity level	−0.016 ± 0.045	0.127	0.722	0.005 [0.000, 0.219]	24
			Relative similarity consistency	−0.056 ± 0.044	1.529	0.216	0.064 [0.000, 0.346]	

## Discussion

In the present study, we did not detect any effects of the level or consistency of female boldness on male mating preference. Females showed stable personality differences in our measure of boldness (with and without controlling for the trial number). Male boldness was only repeatable when controlling for the trial number. In both sexes, the level of boldness increased with the number of times being tested.

To the best of our knowledge, this is the first study testing for male mate choice in *P. pulcher*. Therefore, there is no empirical evidence for the existence of male mate choice in our target species. We had expected male mate choice in *P. pulcher* because there is strong empirical evidence for male choice in a closely related sister species with very similar breeding ecology, *P. taeniatus*. Male *P. taeniatus* choose their mate based on relatedness (Thünken et al., 2011), colouration (Baldauf et al., 2011) and ornamentation (Baldauf et al., 2010). Other recent studies found no support for male mate choice in bi-parental species, namely the convict cichlid, *Amatitlania siquia* (Laubu et al., 2017) and the zebra finch, *Taeniopygia guttata* (Wang et al., 2017; Wang, Forstmeier & Kempenaers, 2017).

In our study, a biological explanation for the lack of male choice could be the existence of typical sex roles during parental care with the male engaging into parental defence behaviours and the female providing direct care. Under this constellation, the effect of female boldness on the performance of maternal care duties may be rather low and might therefore not be very important during mate choice. But, in three-spined sticklebacks, *Gasterosteus aculeatus*, boldness and sociability are negatively correlated (Jolles et al., 2015); possibly suggesting that boldness might also indirectly affect maternal care. Further, a strict parental role allocation defined solely by the sex would not be in line with the female preference for male boldness: female rainbow kribs preferred males of a dis-similar level of boldness indicating parental roles are rather determined by the mate’s personality than by the sex (Scherer, Kuhnhardt & Schuett, 2017a). That is, the shy individual would perform a greater proportion of the direct care while the bold individual would specialize on defence behaviours.

Beside the above biological explanation for the lack of male preference for boldness in our study there are several potentially confounding effects that might have affected our results. First, fish were kept in same-sex family groups. Such holding conditions are required in order to avoid territorial and aggressive behaviour as well as individual differences in reproductive experience. However, these holding conditions led to a heavily biased male sex ratio possibly causing a decline in choosiness. That is because any skew increases direct costs of mate sampling, intra-sexual competition and the risk of ending up unmated for the sex in greater number (Kokko & Mappes, 2005; Dechaume-Moncharmont, Brom & Cézilly, 2016). A biased sex ratio can cause a lack of experience needed to discriminate between potential mates (Rosenqvist & Houde, 1997; Hebets, 2003; Dukas, 2005; Bailey & Zuk, 2008). Both male inexperience and the male-biased sex ratio in stock tanks might have caused the lack of male discrimination between potential mates in the present study. On the other hand, similarly inexperienced females kept under identical holding conditions did show mating preferences for boldness in our female mate choice study (Scherer, Kuhnhardt & Schuett, 2017a).

Second, females were paired up to dyads randomly in regard to their behaviour. This resulted in female dyads being differently contrasted in their level and consistency of boldness, including very poorly contrasted female dyads. However, a removal of poorly contrasted female dyads from the data set did not affect the result of our preference analyses.

Third, in the present study, male repeatability of boldness was unexpectedly low and was only present when accounting for the trial number. Former measurements of male boldness in this species (Scherer, Kuhnhardt & Schuett, 2017a) revealed much higher behavioural stability suggesting a possible noise (e.g., caused by the strong increase of male boldness from the first to the second boldness test) in male behavioural data of this study. If male preference for female boldness is related to male boldness (as expected) a noise in male personality assessment could mask a potential preference for (dis-)similar females.

The increase in the level of male and female boldness with the number of times being tested may indicate habituation to the stimulus (Bell, Hankison & Laskowski, 2009; Nakagawa & Schielzeth, 2010). That is, individuals might get less sensitive to the predator stimulus with time because they have learned from former experiences that it does not pose a threat to them. We emphasize caution in repeatedly using a behavioural assay to measure personality traits. For instance, boldness can hardly be tested over and over again using the same stimulus and procedure without confounding the assessment with habituation. This poses an issue that is important, yet difficult to tackle. Effects of habituation are hard to get rid of; but could be reduced, for example, by modifying the stimulus used between successive measurements and controlling for the number of times being tested in between-individual comparisons.

## Conclusions

Comparing our results to our female mate choice study for boldness (Scherer, Kuhnhardt & Schuett, 2017a) we discover two main differences. First, male behavioural repeatability strongly decreased in the present study compared to our female choice study. Although we are not certain about the reason for the low male repeatability this might be (at least partly) attributed to a follow-up effect of behavioural habituation to the stimulus. Second, while female mate choice was affected by an interplay between male and female behaviour, we did not detect any effects of female boldness on male choice. Sexual selection might act differently on male and female boldness because boldness may affect male (territory defence) but not female (direct offspring care) parental care behaviour. On the other hand, (dis-)assortment shown by the females indicates mutual mate assessment (Johnstone, 1997). The causality in male–female preference mismatch remains unclear. Therefore, further research is needed to test how the interplay between parental personalities and offspring care is linked to an individual’s fitness in order to shed light on the driving evolutionary mechanisms that form stable personality variation in bi-parental species.

##  Supplemental Information

10.7717/peerj.5373/supp-1 Data S1 Behavioural raw data obtained during boldness tests

10.7717/peerj.5373/supp-2 Data S2 Behavioural data obtained during mate choice trials

## Additional Information and Declarations

### Data Availability

The following information was supplied regarding data availability: The raw data are provided in a Supplemental File.

## References

[ref-1] Amundsen T, Forsgren E (2001). Male mate choice selects for female coloration in a fish. Proceedings of the National Academy of Sciences of the United States of America.

[ref-2] Arnaud L, Haubruge E (1998). Mating behaviour and male mate choice in *Tribolium castaneum* (Coleoptera, Tenebroinidae). Behaviour.

[ref-3] Bailey NW, Zuk M (2008). Acoustic experience shapes female mate choice in field crickets. Proceedings of the Royal Society B.

[ref-4] Baldauf SA, Bakker TC, Herder F, Kullmann H, Thünken T (2010). Male mate choice scales female ornament allometry in a cichlid fish. BMC Evolutionary Biology.

[ref-5] Baldauf SA, Bakker TCM, Kullmann H, Thünken T (2011). Female nuptial coloration and its adaptive significance in a mutual mate choice system. Behavioral Ecology.

[ref-6] Bateman JA (1948). Intra-sexual selection in *Drosophila*. Heredity.

[ref-7] Bell AM, Hankison SJ, Laskowski L (2009). The repeatability of behaviour: a meta-analysis. Animal Behaviour.

[ref-8] Bierbach D, Sassmannshausen V, Streit B, Arias-Rodriguez L, Plath M (2013). Females prefer males with superior fighting abilities but avoid sexually harassing winners when eavesdropping on male fights. Behavioral Ecology and Sociobiology.

[ref-9] Bonduriansky R (2001). The evolution of male mate choice in insects: a synthesis of ideas and evidence. Biological Reviews.

[ref-10] Bonduriansky R, Brooks RJ (1998). Male antler flies (*Protopiophila litigata*; Diptera: Piophilidae) are more selective than females in mate choice. Canadian Journal of Zoology.

[ref-11] Caballero-Mendieta N, Cordero C (2013). Male mating costs in a butterfly that produces small ejaculates. Physiological Entomology.

[ref-12] Cantoni D, Brown RE (1997). Paternal investment and reproductive success in the California mouse, *Peromyscus californicus*. Animal Behaviour.

[ref-13] Chira A (2014). How does parental personality influence offspring quality in animals?. Annals of Forest Research.

[ref-14] David M, Pinxten R, Martens T, Eens M (2015). Exploration behavior and parental effort in wild great tits: partners matter. Behavioral Ecology and Sociobiology.

[ref-15] Dechaume-Moncharmont F-X, Brom T, Cézilly F (2016). Opportunity costs resulting from scramble competition within the choosy sex severely impair mate choosiness. Animal Behaviour.

[ref-16] Dechaume-Moncharmont FX, Cornuau JH, Keddar I, Ihle M, Motreuil S, Cézilly F (2011). Rapid assessment of female preference for male size predicts subsequent choice of spawning partner in a socially monogamous cichlid fish. Comptes Rendus Biologies.

[ref-17] Doutrelant C, McGregor PK (2000). Eavesdropping and mate choice in female fighting fish. Behaviour.

[ref-18] Dukas R (2005). Learning affects mate choice in female fruit flies. Behavioral Ecology.

[ref-19] Edward DA, Chapman T (2011). The evolution and significance of male mate choice. Trends in Ecology & Evolution.

[ref-20] Gross MR, Sargent RC (1985). The evolution of male and female parental care in fishes. American Zoologist.

[ref-21] Hebets EA (2003). Subadult experience influences adult mate choice in an arthropod: exposed female wolf spiders prefer males of a familiar phenotype. Proceedings of the National Academy of Sciences of the United States of America.

[ref-22] Herdman EJE, Kelly CD, Godin J-GJ (2004). Male mate choice in the guppy (*Poecilia reticulata*): do males prefer larger females as mates?. Ethology.

[ref-23] Itzkowitz M (1984). Parental division of labor in a monogomous fish. Behaviour.

[ref-24] Jaeger B (2016). https://CRAN.R-project.org/package=r2glmm.

[ref-25] Johnstone RA (1997). The tactics of mutual mate choice and competitive search. Behavioural Ecology and Sociobiology.

[ref-26] Jolles JW, Fleetwood-Wilson A, Nakayama S, Stumpe MC, Johnstone RA, Manica A (2015). The role of social attraction and its link with boldness in the collective movements of three-spined sticklebacks. Animal Behaviour.

[ref-27] Kokko H, Jennions M (2003). It takes two to tango. Trends in Ecology & Evolution.

[ref-28] Kokko H, Mappes J (2005). Sexual selection when fertilization is not guaranteed. Evolution.

[ref-29] Kontiainen P, Pietiäinen H, Huttunen K, Karell P, Kolunen H, Brommer JE (2009). Aggressive Ural owl mothers recruit more offspring. Behavioral Ecology.

[ref-30] Kralj-Fišer S, Sanguino Mostajo GA, Preik O, Pekár S, Schneider JM (2013). Assortative mating by aggressiveness type in orb weaving spiders. Behavioral Ecology.

[ref-31] Laubu C, Dechaume-Moncharmont FX, Motreuil S, Schweitzer C (2016). Mismatched partners that achieve postpairing behavioral similarity improve their reproductive success. Science Advances.

[ref-32] Laubu C, Schweitzer C, Motreuil S, Louâpre P, Dechaume-Moncharmont F-X (2017). Mate choice based on behavioural type: do convict cichlids prefer similar partners?. Animal Behaviour.

[ref-33] Lavery RJ, Reebs SG (1994). Effect of mate removal on current and subsequent parental care in the convict cichlid (Pisces: Cichlidae). Ethology.

[ref-34] Marconato A, Bisazza A, Fabris M (1993). The cost of parental care and egg cannibalism in the river bullhead, *Cottus gobio* L. (Pisces, Cottidae). Behavioural Ecology and Sociobiology.

[ref-35] McKaye KR, Murry BA (2008). Sex role differentiation in brood defense by Nicaraguan cichlid fish, *Amphilophus xiloanensis*. Caribbean Journal of Science.

[ref-36] Montiglio PO, Wey TW, Chang AT, Fogarty S, Sih A (2016). Multiple mating reveals complex patterns of assortative mating by personality and body size. Journal of Animal Ecology.

[ref-37] Mutzel A, Dingemanse NJ, Araya-Ajoy YG, Kempenaers B (2013). Parental provisioning behaviour plays a key role in linking personality with reproductive success. Proceedings of the Royal Society B.

[ref-38] Nakagawa S, Schielzeth H (2010). Repeatability for Gaussian and non-Gaussian data: a practical guide for biologists. Biological Reviews.

[ref-39] Olsson M (1993). Male preference for large females and assortative mating for body size in the sand lizard (*Lacerta agilis*). Behavioural Ecology and Sociobiology.

[ref-40] Olsson M, Madsen T, Shine R (1997). Is sperm really so cheap? Costs of reproduction in male adders, *Vipera berus*. Proceedings of the Royal Society B.

[ref-41] R Core Team (2017). http://www.R-project.org/.

[ref-42] Reinhold K, Schielzeth H (2015). Choosiness, a neglected aspect of preference functions: a review of methods, challenges and statistical approaches. Journal of Comparative Physiology. A, Neuroethology, Sensory, Neural, and Behavioral Physiology.

[ref-43] Rosenqvist G, Houde A (1997). Prior exposure to male phenotypes influences mate choice in the guppy, *Poecilia reticulata*. Behavioural Ecology.

[ref-44] Royle NJ, Schuett W, Dall SRX (2010). Behavioral consistency and the resolution of sexual conflict over parental investment. Behavioral Ecology.

[ref-45] Royle NJ, Smiseth P, Kölliker M (2012). The evolution of parental care.

[ref-46] Scherer U, Godin JGJ, Schuett W (2017b). Validation of 2D-animated pictures as an investigative tool in the behavioural sciences—a case study with a West African cichlid fish, *Pelvicachromis pulcher*. Ethology.

[ref-47] Scherer U, Kuhnhardt M, Schuett W (2017a). Different or alike? Female rainbow kribs choose males of similar consistency and dissimilar level of boldness. Animal Behaviour.

[ref-48] Scherer U, Kuhnhardt M, Schuett W (2018). Predictability is attractive: female preference for behaviourally consistent males but no preference for the level of male aggression in a bi-parental cichlid. PLOS ONE.

[ref-49] Schlupp I, Marler C, Ryan MJ (1994). Benefit to male sailfin mollies of mating with heterospecific females. Science.

[ref-50] Schlupp I, Waschulewski M, Ryan MJ (1999). Female preferences for naturally-occurring novel male traits. Behaviour.

[ref-51] Schneider CA, Rasband WS, Eliceiri KW (2012). NIH Image to ImageJ: 25 years of image analysis. Nature Methods.

[ref-52] Schuett W, Dall SRX, Royle NJ (2011b). Pairs of zebra finches with similar ‘personalities’ make better parents. Animal Behaviour.

[ref-53] Schuett W, Godin JGJ, Dall SRX (2011a). Do female zebra finches, *Taeniopygia guttata*, choose their mates based on their ‘personality’?. Ethology.

[ref-54] Schuett W, Nava TF, Rahmlow N, Scherer U (2017). Artificial Visible Implant Elastomer (VIE) tags of different colour and symmetry do not influence mate choice in a cichlid. Behaviour.

[ref-55] Schuett W, Tregenza T, Dall SRX (2010). Sexual selection and animal personality. Biological Reviews.

[ref-56] Steinhart GB, Sandrene ME, Weaver S, Stein RA, Marschall EA (2004). Increased parental care cost for nest-guarding fish in a lake with hyperabundant nest predators. Behavioral Ecology.

[ref-57] Stoffel MA, Nakagawa S, Schielzeth H (2017). rptR: repeatability estimation and variance decomposition by generalized linear mixed-effects models. Methods of Ecology and Evolution.

[ref-58] Svensson I (1988). Reproductive costs in two sex-role reversed pipefish species (*Syngnathidae*). Journal of Animal Ecology.

[ref-59] Teyssier A, Bestion E, Richard M, Cote J (2014). Partners’ personality types and mate preferences: predation risk matters. Behavioral Ecology.

[ref-60] Thünken T, Bakker TC, Baldauf SA, Kullmann H (2007). Active inbreeding in a cichlid fish and its adaptive significance. Current Biology.

[ref-61] Thünken T, Baldauf SA, Kullmann H, Schuld J, Hesse S, Bakker TCM (2011). Size-related inbreeding preference and competitiveness in male *Pelvicachromis taeniatus* (Cichlidae). Behavioral Ecology.

[ref-62] Trivers RL, Campbell B (1972). Parental investment and sexual selection. Sexual selection and the descent of man, 1871-1971.

[ref-63] Wang D, Forstmeier W, Kempenaers B (2017). No mutual mate choice for quality in zebra finches: time to question a widely held assumption. Evolution.

[ref-64] Wang D, Kempenaers N, Kempenaers B, Forstmeier W (2017). Male zebra finches have limited ability to identify high-fecundity females. Behavioral Ecology.

[ref-65] Wedell N, Gage MJG, Parker GA (2002). Sperm competition, male prudence and sperm-limited females. Trends in Ecology & Evolution.

[ref-66] Welke KW, Zimmer SM, Schneider JM (2012). Conditional monogyny: female quality predicts male faithfulness. Frontiers in Zoology.

[ref-67] Wiegmann DD, Angeloni LM, Seubert SM, Wade JG (2013). Mate choice decisions by searchers. Current Zoology.

[ref-68] Wilson DS, Clark AB, Coleman K, Dearstyne T (1994). Shyness and boldness in humans and other animals. Trends in Ecology & Evolution.

[ref-69] Witte K, Godin J-GJ (2010). Mate choice copying and mate quality bias: are they different processes?. Behavioral Ecology.

[ref-70] Wong BB, Jennions MD (2003). Costs influence male mate choice in a freshwater fish. Proceedings of the Royal Society B.

